# Are in‐person scientific conferences dead or alive?

**DOI:** 10.1096/fba.2020-00139

**Published:** 2021-02-20

**Authors:** Nick Dua, Mattias Fyrenius, Deborah L. Johnson, Walter H. Moos

**Affiliations:** ^1^ Keystone Symposia Silverthorne CO USA; ^2^ Bonnier Carlsen Stockholm Sweden; ^3^ Baylor College of Medicine Houston TX USA; ^4^ University of California San Francisco San Francisco CA USA; ^5^ Pandect Bioventures South San Francisco CA USA

**Keywords:** conference, COVID‐19, face‐to‐face, in‐person, meeting, scientific communication/interchange, symposia, virtual

## Abstract

Given the disruption caused by the COVID‐19 pandemic, life as we knew it has been turned upside down, but the need for science to go on has never been stronger. In the realm of scientific conferences, with the requirement for social distancing, the importance of wearing face coverings, and travel restrictions, only virtual meetings have been possible during the pandemic. But many are asking: What is the new post‐pandemic normal likely to be? Do we still want to have in‐person meetings when the restrictions are eased? Assuming we do, when will they be possible again, and under what conditions? Regardless of what the benefits of virtual symposia might be, are they here to stay? These questions, and many more that are being asked around the world today, are the subject of this perspective. Herein, we attempt to provide useful context and insight into where scientific meetings have been, where they are today, where they are going, and how they will get there. Our conclusion is that the pandemic has created an accelerated opportunity to make the world of future scientific conferences better in a “both/and” collaborative in‐person/virtual scenario, not the more limited “pick one” choice.

## 1 INTRODUCTION

**FIGURE 1:**
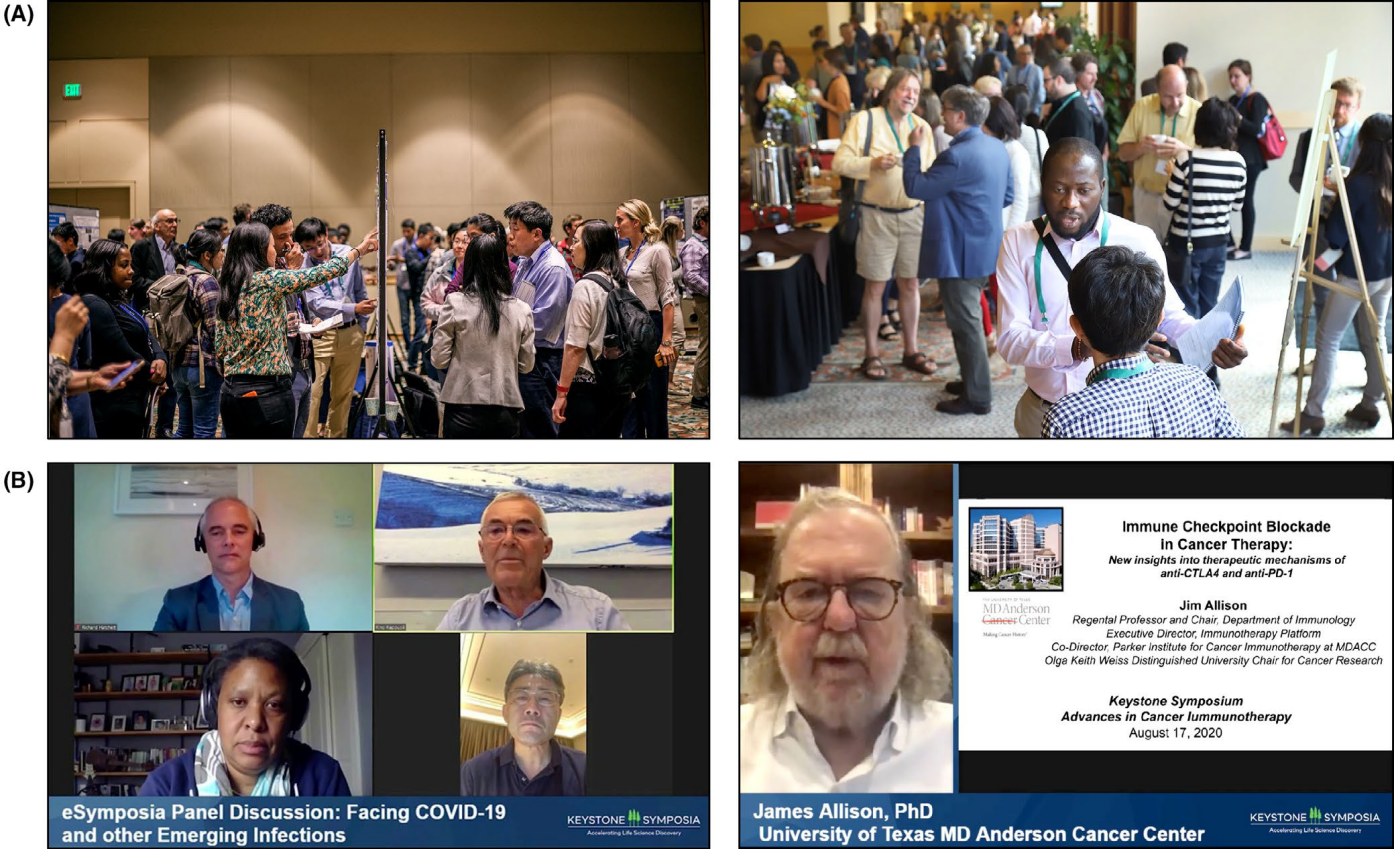
Which would you prefer to attend, A and/or B? (A) How a poster session and a break during a conference looked before the COVID‐19 pandemic? (B) How virtual conferences look during the COVID‐19 pandemic in 2020?

In‐person scientific conferences are dead—long live in‐person conferences! But virtual symposia are here to stay. Those two sentences describe the conclusions of this article well, and Figure 1 asks the key question. Maybe you were not expecting epanalepsis in a scientific journal, but there it is, a repetition and seeming contradiction of the first part of this article's opening sentence at the end of the same sentence. This phrasal construction first arose hundreds of years ago in the succession of monarchs in Europe and has subsequently been utilized to emphasize the replacement, resumption, or succession of many things. Unusual, yes, yet the unusual is to be expected these days, given how the pandemic has turned so much of life upside down. The new normal is changing everything, including the tried‐and‐true symposium formula for in‐person knowledge transfer, scientific debate, and chance interactions that help to fuel innovation in many fields, including our focus, life sciences, especially human biology and medicine.

The novel severe acute respiratory syndrome coronavirus‐2 (SARS CoV‐2) and the consequent coronavirus infectious disease first reported in 2019 (COVID‐19) have rendered in‐person meetings and other face‐to‐face interactions awkward at times, and often unwise. As of 22 January 2021, during a major uptick in COVID‐19 in many countries around the world, with nearly 100 million global cases, over 2 million global deaths, and almost 25 million and 415,000 cases and deaths, respectively, in the United States (https://coronavirus.jhu.edu/map.html). Life has changed immeasurably for so many people. Thus, it should be no surprise that in the realm of scientific conferences, given the need for social distancing, the importance of wearing face coverings, and travel restrictions, many are asking:

- What is the new post‐pandemic normal likely to be?
- How much of what we are forced to do now will we still do when we are no longer so constrained?
- Do we still need in‐person meetings when the restrictions are eased? (Yes!)
- Assuming we do leave this transient state and return to in‐person conferences, how soon will they be possible again, and under what conditions?
- What are the benefits of virtual meetings, alone and in combination with in‐person meetings, and are they here to stay?

These questions, and many more that are being asked around the world today, are the subject of this perspective. We do expect to return from the shock^1^ of the new normal to the old normal when it is safe again to do so, but after we reach that inflection point we should strive to keep the new approaches that enhance productive interchange between scientists. Our bottom line: We believe that the pandemic has created an accelerated opportunity to make the world of future scientific conferences better in a “both/and” collaborative in‐person/virtual scenario, not the more limited “pick one” choice.

## 2 THE PAST

In‐person scientific meetings have been a preferred mode to share new data and perspectives and to stimulate healthy discussion and debate since mankind first gathered to discuss their existence and the world around them. Many in‐person formats have made up such events in modern days, usually including some combination of formal oral presentations, with keynote speeches from luminaries to open and/or to conclude a symposium, panel and roundtable discussions, poster sessions, and workshops. Typically, group meals and mixers would also be scheduled, and in some cases, portions of the day would be set aside for unscheduled activities per personal preferences, such as hiking, skiing, swimming, or taking advantage of other local leisure activities. These activities often provided additional opportunities for interactions between attendees. Alternatively, some attendees might keep to themselves, catching up on emails, writing papers or proposals, preparing slides for talks, getting some much‐needed sleep, or just sitting outside in the sun in warm settings or around an outdoor fire pit or an indoor fireplace if cold. See Figure 1.

Some of the best meetings are in our opinion those run by nonprofit organizations, often held in pleasant settings that facilitate informal discourse, providing opportunities for speakers to present unpublished data and for serendipitous interactions to occur that catalyze new insights, discoveries, and professional relationships. Certain meeting organizations have a long history of providing forums for early presentations around major scientific advances, such as the link between AIDS and the retrovirus now known as HIV. For instance, in the United States, nonprofits like the Gordon Research Conferences and Keystone Symposia (begun originally as the ICN‐UCLA Symposia on Molecular Biology) date back to 1931 and 1972, respectively. A few examples of important nonprofit organizations that provide scientific conferences of similar size in attendance (generally <500) in the life sciences are provided in Table 1, which is focused on Keystone Symposia‐like entities. Readers should note that there are hundreds of national and international meetings not listed here, organized by a host of learned societies and dedicated disease associations, which collectively make up a large proportion of bioscience meetings.

**TABLE 1:** A few examples of leading nonprofit conferences covering life sciences

American Association for Cancer Research Meetings (AACR) https://www.aacr.org/professionals/meetings/	Cold Spring Harbor Conferences meetings.cshl.edu	Gordon Research Conferences www.grc.org
Federation of American Societies for Experimental Biology Meetings www.faseb.org/Science‐Research‐Conferences	Keystone Symposia www.keystonesymposia.org/ks/online	EMBO/EMBL Symposia www.embo‐embl‐symposia.org

Other meeting venues that have varying levels of scientific ambitions without being traditionally scientific include the Nobel Prize Dialogue (https://www.nobelprize.org/nobel‐prize‐dialogue/), Sci Foo Camp (https://www.digital‐science.com/events/science‐foo‐camp/), TED Talks (https://www.ted.com/talks), and “unconferences” (https://www.forbes.com/sites/rebeccabagley/2014/08/18/how‐unconferences‐unleash‐innovative‐ideas/?sh=58aa022c645b). These, like most or all successful meetings, address common psychological needs, which in a simple rendering must attend to feelings of choice, competence, and connectivity. Some organizations, like the New York Academy of Sciences (https://www.nyas.org/events/), provide written commentaries on their conferences.^2^

As an aside, albeit of less relevance for the type of scientific interchange we focus on while still being worth mentioning, physical meetings such as global conferences (e.g., the World Economic Forum in Davos, Art Biennales, and various world expos) have been extremely valuable from a public relations perspective. These gatherings, including those produced for Nobel Prizes, have as a key purpose the generation of media and public interest in the topics at hand. Thus, journalists tend to appreciate having both many key people gathered in one space as well as having a context in which to write about them. The desired outcome is much easier to achieve with a physical meeting.

What about virtual scientific meetings in the “old days”? In fact, online and videoconference‐based meetings have taken advantage of many stages of technological progress. One of the major steps forward can be traced back to 1968 and the so‐called “Mother of All Demos” (https://www.sri.com/case‐studies/how‐a‐90‐minute‐presentation‐became‐the‐catalyst‐to‐the‐modern‐world‐of‐personal‐computing/). In a tour de force, researchers at the Stanford Research Institute (SRI) displayed a range of new engineering tools, including the computer mouse, a personal computer, and live video transmission. The origin of today's internet traces back to the late 1960s too, as part of a wide‐area networking project known as “ARPANET,” sponsored by the US government's Advanced Research Projects Agency (ARPA, now known as DARPA), which included SRI, the University of California Los Angeles, the University of California Santa Barbara, and the University of Utah (https://www.sri.com/hoi/arpanet/).

However, it was not until the 1990s that videoconferencing started to become more commonplace, principally in corporate settings given the expense of the equipment and the cost of dedicated communication lines. This was followed, principally after the turn of the millennium, by internet‐based virtual meetings, which have become progressively more popular. Both older and newer internet‐based conferencing technologies, for example, Skype (now more or less integrated into Microsoft Teams) and Zoom, respectively, were ready and waiting when the pandemic took charge of our lives.

## 3 THE PRESENT

It is imperative that science goes on, but what is possible in the middle of a pandemic? A recent editorial^3^ focused on three things:

- “democratizing science”;
- “balancing ease of access versus unique benefits of immersive events”; and
- “fostering personal connections and catalyzing collaboration.”

But how does one “go virtual” and navigate the myriad complexities of technology and human interactions in today's environment? Indeed, there are many challenges to consider (Table 2).

**TABLE 2:** Some challenges in doing science and holding meetings in the midst of a pandemic^4^

Disease outbreaks and lockdowns	Internet bandwidth limitations and computer crashes	Lack of clear national pandemic strategies in many countries
More limited support services	Quarantines	Restricted travel
Social distancing	Stress in general	Transition to new online tools that may still have bugs
Disruption of clinical trials	Limited access to academic research laboratories	Difficulty in obtaining needed resources for conducting research

Conference providers who convene in‐person meetings have been forced to rethink their offerings with lightning speed, a real test of an organization's agility. We are reminded of an old grammatically incorrect but memorable Apple Computer ad, “Think different,” Keystone Symposia and other groups have had to think differently in order to flip their models from most or all meetings being in‐person to all meetings being virtual, at least until the pandemic is brought under control. It is not clear at the time of this writing when in‐person meetings will resume, though as of late 2020 some groups were planning to restart as early as 2Q 2021, such as the American College of Cardiology (ACC.21; https://accscientificsession.acc.org/?_ga=2.197329961.702945313.1604789543‐1370724806.1604340463). It is unknown how much of an impact the apparent recent progress with vaccines and therapeutics will have, or how soon, but the reports are promising and a new national strategy should help (see: https://www.whitehouse.gov/wp‐content/uploads/2021/01/National‐Strategy‐for‐the‐COVID‐19‐Response‐and‐Pandemic‐Preparedness.pdf).

Catalyzed by the pandemic, conference organizations have had to accelerate their digital strategies to support the business and to keep pace with the competition. With a growing number of off‐the‐shelf internet‐based meeting products as well as with the improvement and proliferation of content delivery networks, virtual events and other digital experiences have become an accepted reality that will surely endure and be part of our post‐pandemic lives.

These technologies, platforms, and services have proliferated to meet the increased demand from conference organizers. The expectations have gone beyond information dissemination and have evolved to community engagement and collaboration. It is not enough to be able to present the latest research. Today's scientific conference organizers must recreate experiences that emulate the serendipity of an in‐person meeting together with its opportunities for networking, collaboration, and ideation. Several compelling products, platforms, and strategies have emerged and have become available to conference organizers. See Figure 1 for a peek at what virtual conferences look like today.

### 3.1 Engagement is key

Digitell has been the Keystone Symposia digital media partner since 2015. They host our Virtual Keystone Symposia VKS platform, where we engage the global scientific community with ePanel events, scientific talks (SciTalks), and other interesting content inspired by our meetings. (See, e.g., http://bit.ly/VKSdrugdisc.) When we launched our eSymposia virtual meeting series, Digitell provided a suite of tools designed to optimize the audience experience, including the following.

- Virtual Poster Booths: A feature within the eSymposia platform where we showcase abstracts, ePosters, and pre‐recorded presentations, as well as providing the ability to live chat with presenters during designated times. We are currently working with Digitell as well as other providers to produce live video chats to increase engagement with poster presenters.
- 1:1 Connect: The ability for eSymposia attendees to have 1‐on‐1 video conversations with one another.
- Interactive Forums: During our plenary sessions, each speaker is afforded 5–10 min for a live question and answer period with the audience. Often this is not enough time to cover all questions. The interactive forums provide an additional venue to engage with speakers and continue the interaction.
- Breakout Sessions: Between sessions at our eSymposia events, we often organize “Breakout Zoom Rooms” (e.g., Meet the Editors and Career Roundtables). These are smaller engagements designed to provide mentorship and career advice to early career attendees of the event.

More enhancements are planned to improve both audience engagement as well as real‐time management of these virtual events.

### 3.2 Additional conference platforms

Digitell is just one of a host of virtual conference platforms that have been taking unique paths in their service offerings. A couple of other examples are noted below.

- Remo (https://remo.co): A different approach to virtual events. Using a 2‐dimensional floorplan interface, they have incorporated video interactions for both large audience presentations as well as small networking engagements.
- Bevy (https://www.bevy.com): Built with a community in mind, not just virtual event management. Included are breakout rooms, interactive chats, and networking rooms.

### 3.3 Whatever happened to virtual reality?

The subject of 1980s and 1990s science fiction books and movies saw us interacting with virtual worlds that provided promise as well as a cautionary tale about these augmented experiences. Bolstered by the video game industry, virtual reality (VR) has experienced a resurgence in some settings.

Platforms like VirBELA (https://www.virbela.com) and Sinespace Breakroom (https://sine.space/breakroom) bring communities together in three‐dimensional virtual spaces, where they host conferences and provide both structured and unstructured programing that drives attendees to explore the virtual world and to engage randomly with other attendees. Exhibition halls, theaters, and casual open spaces dot the virtual landscape. Through their suite of video conferencing and media sharing tools, participants are able to experience the presentations and to interact with fellow delegates.

However, we must voice a word of caution regarding VR as a near‐term media form. It is good to consider VR as a potential component in the future of scientific meetings, but there has been a level of hype around such technologies that appears to have dissipated in recent times. When and if VR will change the world remains an open question.

### 3.4 The show must go on—media approaches to technical endeavors

One consideration that is sometimes overlooked in producing a virtual event is the fact that these engagements are media broadcast productions where the preparation, pacing, structure, and length are all carefully orchestrated to make certain that the entire event is of high quality, user‐friendly to participants, and engaging for all. “Tech checks” with the speakers, moderators, and other presenters are highly valued to ensure that everyone is prepared and knows what to anticipate. Community facilitators are active throughout the event to make sure that engagement is encouraged and wayfinding is constantly promoted so that everyone knows what to expect at any given time. The “hosts” (organizers and moderators) have a script to follow and are constantly guided to “keep the show moving.”

With events and meetings becoming more screen‐based, organizers of scientific meetings need to think more like media organizations. They should ask, “What is the best way to engage an audience that is not physically present but instead is sitting in front of a screen?” The dramaturgy necessary for a successful event is likely to change to something more akin to a television production, albeit with interactive dimensions. The uniqueness of the live experience will change in the same way that a live theater performance is something very different from a film. But the lasting value of e‐symposia is that a recorded meeting with a screen‐first approach will for sure live longer on‐demand. After all, it is meant to be watched on a screen, live or on‐demand, thus achieving greater and greater reach and impact over time.

Virtual meetings do indeed provide a number of advantages, alone or in combination with in‐person events (Table 3), which may suitably counterbalance potential challenges (Table 4). It is worth calling out the carbon footprint element of traveling to meetings. We expect that a climate sustainability argument will be key for both younger and older generations. Thus, it will be difficult to argue in favor of flying people across the globe just for “short meetings.”

**TABLE 3:** Potential advantages of virtual symposia

Lower cost for registration	More people can attend from around the world	Lower carbon footprint
New collaboration technologies	More accessible to individuals with disabilities	Easier for people with dependents (including children) to attend
Maybe less intimidating for students to engage with leaders in their fields	Mitigates the risk of contracting COVID−19 and other illnesses	Eliminates travel time and expenses

**TABLE 4:** Possible issues with virtual symposia

Can organizers and providers make the economics work?	Internet connections can be unstable, especially for high bandwidth needs	Poster sessions may struggle to achieve the richness of in‐person discussions
Less serendipity?	Large time zone differences if participating in real time	Lack of complete immersion in the conference

Given the unpredictability of force majeure events like the current pandemic, what does a conference organization need to consider when canceling or rescheduling in‐person meetings, or when repositioning from in‐person to virtual meetings or vice versa? A few questions to ask yourself can be found in Table 5. In our view, keeping a mindset of always doing what's right is the way to think this through.

**TABLE 5:** What organizers might consider when canceling or rescheduling in‐person meetings, or when repositioning from in‐person meetings to virtual conferences or vice versa

What is the most responsible thing to do?	Do you have cancellation insurance at the event site?	Do you have travel cancellation insurance?
Will people attend?	Are the organizers willing?	Do you have sufficient financial support to at least break even?
How far in advance do you need to finalize the event?	Will speakers be willing to present their latest (often unpublished) data?	Can the meeting be held safely?
Are there any travel restrictions and quarantine requirements?	Are there competing meetings that need to be considered?	The unknown…

In our experience in 2020, there was an initial reluctance on the part of some organizers to reconvene their meetings as virtual events. However, with the unrelenting progression of the pandemic, many came to realize that it was critical for the scientific community to remain active and engaged and that virtual events could greatly extend the reach to early career investigators and those from low‐and‐middle‐income countries who would not be able to attend an in‐person meeting. During the pandemic, the attendance for most of the virtual meetings we have been involved with has exceeded what would have been expected for in‐person attendance in the past.

## 4 THE FUTURE

We believe that a melding of virtual and in‐person meetings has the potential to be the best of all possible worlds. The electronic medium presents certain advantages for sure. In‐person meetings do too. Those who meld the two well should excel.

We expect the technologies that facilitate virtual meetings to continue to advance. Like stadium concerts surviving streaming music, in‐person meetings are here to stay and will be a willing partner to share the stage with their virtual counterparts. They are simply different experiences and address different needs for specific audiences. In many instances, they will complement each other favorably in the form of hybrid meetings.

Having mentioned live streaming in the context of rock music and stadium events, we would be remiss if we were not to consider the arts more broadly. As the pandemic comes under control, we expect Broadway will reopen with live theatre despite the success of Disney's Hamilton filming, the Met will reopen with live opera despite years of professional live streaming to movie theatres around the country, and symphonies in every city predict a robust return to live audiences. Moreover, there was a time when there was a reluctance to televise live professional baseball and football games because of the fear that doing so would lead to the demise of stadium attendance.

Interestingly enough, in analogy, live concerts have not only survived streaming services and digital music, but they have actually flourished. This is true both in general, in terms of numbers of users and economic importance for the industry, and in particular, for the artists. It is said that in the music business you used to tour to sell albums; now you stream your music to sell tickets for your concerts. This is an argument for the blended strategy that we believe will be the end result of the current evolution of scientific meetings.

We complete the current context with reference to educational institutions, particularly universities and medical schools. Now virtual in many settings, teaching via online lectures, even with class interruptions, questions, and discussion, is in many ways analogous to scientific conferences. While one might expect more virtual online classes and lectures in the future, no one is predicting the end of college on‐campus experiences and learning. All of these examples are relevant to the case we are trying to make.

### 4.1 High expectations—high anxiety

By the time we start reconvening in‐person conferences, a good portion of the community will have attended one or more virtual scientific conferences. As such, they will have specific expectations on what makes a good digital experience. We expect that interaction and engagement will prove to be two of the most important features expected in future virtual meetings. Conference organizers are currently in different stages of experimentation with interactivity. However, with texting, direct messaging, voice assistants, and face‐time tools already available and part of consumers’ daily lives, there will be aspects of virtual engagement that attendees will not only expect to be part of the experience, they will expect it to work flawlessly.

Key challenges for virtual events circle around the elements of interaction, engagement, and serendipity. Even if virtual technologies improve and address these most important elements better over time, they will probably never be quite as good as in‐person interactions. That in itself is an answer to why blended meeting formats will be the best way forward. To paraphrase Shakespeare, “Such stuff that in‐person conferences are made on” will likely never be achieved online only, at least not in our lifetimes.

As scientific meetings evolve, we expect some to begin to react with, “Your virtual event is how long?!” That is, once everyone gets to the other side of the present “lockdown” existence and we resume our “new old normal” (at work, at play, and with family), a virtual conference lasting three full days might be too much to ask of most individuals. As one of our colleagues described a short virtual meeting that he experienced, a “high‐octane half‐day meeting” might suffice for future engagements, or a full day's worth of content spread out over multiple days. With our tradition (and obsession) with analytics, feedback, and observations, we should have a good idea what those thresholds will be once we complete this season's series of virtual meetings.

### 4.2 Virtual reality realized?

With the gaming industry paving the way, further developments in virtual and augmented reality are beginning to show promise. The commitment of technology giants like Facebook and Apple to Oculus and Glass, respectively, may enable attendees to attend and interact in a VR conference in a not‐so‐distant future. While we voiced a note of caution earlier about VR as a future media form, innovative new technologies have a way of sneaking up on what is today the art of the impossible, so stay tuned.

Some have suggested that virtual meetings have become so popular that there will be a permanent shift away from in‐person events (https://www.medscape.com/viewarticle/939403). We say, “Not so fast.” But while we believe that in‐person meetings will eventually return in force, there is no question that virtual meetings are here to stay, both alone and in combination with in‐person settings once they are possible again.

When does the future start? Since March 2020, numerous scientific conferences have been cancelled. This includes various biomedical events organized by national and international academies, associations, and societies, such as the AACR April 2020 meeting (https://www.medscape.com/viewarticle/926116). Keystone Symposia canceled half of their in‐person meetings in the Spring of 2020. The majority of Keystone Symposia in‐person meetings that were scheduled from Fall 2020 through June 2021 will be reconvened as virtual eSymposia events. With the anticipation that in‐person meetings will resume by 2022, how might we best leverage both in‐person and virtual components into our conferences?

As we move forward through the evolving situation with COVID‐19, we believe it is important to develop various options for conferences that minimize cost and maximize benefit while adhering to local health guidelines. Convening in‐person or virtual‐only events, or a hybrid of both, will largely depend on the anticipated attendance numbers, the topics, and the geographic areas where the science is represented. For example, in the case of a meeting that attracts high numbers of attendees and cannot always accommodate everyone wanting to attend in‐person, a hybrid approach with both virtual and in‐person components could ensure greater access. By contrast, for those meetings that convene emerging topics, in‐person events provide meaningful engagement of nascent scientific communities and may not be as well served by a virtual option. However, to increase awareness of these relatively small new meetings, a short virtual “preview” event might ideally be convened prior to the in‐person event. When we are able to convene in‐person meetings again, there are a number of issues (given in Table 6) that will determine whether a hybrid and/or an in‐person meeting is the best approach.

**TABLE 6:** Considerations for conducting hybrid virtual/in‐person meetings

What is the cost of adding a virtual component?	Is there sufficient demand for a virtual component?	Will a virtual option cannibalize in‐person meeting attendance?
Will a virtual option provide increased access to the global community and early‐career investigators?	Is it important that the majority of organizers and speakers attend the in‐person meeting?	Should the virtual component be live or prerecorded?

Let us assume that in‐person conferences will eventually be back in force. What does one need to prepare for to resume in‐person meetings responsibly? Clearly a number of health and safety matters must be considered for the foreseeable future. In Table 7, we provide a list of possible considerations based on a number of assumptions that no one can fully predict at this juncture. There is little question in our minds that we will phase into a vaccine‐driven herd immunity scenario rather than simply assuming that the pandemic is over. That is, we do not expect immediate normalcy. Hence, many if not all of these precautions will be necessary for some period of time, but everything will need to be monitored and re‐evaluated frequently as in‐person meetings resume. These precautions are not likely to be needed forever, but no one knows what the timeframe will be.

**TABLE 7:** Selected health and safety considerations for resumption of in‐person symposia

Adequate air flow and upgraded filtration systems	Box lunches but no buffet or other food sharing areas	Contactless systems wherever possible
Indoor/outdoor venues kept below normal occupancy	Face covering requirements with spare face coverings available	Medical professionals available onsite
More frequent and more extensive cleaning schedules	Proof of recent negative test for SARS CoV−2	Ready access to hand sanitizers, soap, and water
Significant signage and other reminders	Social distancing including partitions where needed	Sufficient local hospital capacity
Unidirectional flow of attendees	Zero tolerance for safety infractions	Reschedule (or go virtual or cancel) if local/regional disease surges

## 5 CONCLUDING REMARKS

The role of technologies in the evolution of professional conferences should not be underestimated. There is no doubt that new technologies will play a greater and greater role in the future, and we believe that the inevitable transition where virtual meetings play a more or less equally important role in scientific discourse has been accelerated by the pandemic. The future is now, but the past will return, and both will evolve, together.

Once they can be reconvened, in‐person meetings will for the foreseeable future provide a richer, easier experience with more chance interactions. These somewhat random interactions could catalyze the next advance in biomedical research that saves the lives of countless sick patients, and every life saved is a miracle—the sooner the better! Less dramatic, but still important, a happenstance introduction of two people at an in‐person conference could launch a student's life science career. Let us make this personal. That student's work could ultimately save your life or save the planet we live on from the ravages of climate change.

In‐person conferences are dead—long live in‐person conferences! But, as we chart the future of scientific interchange, we should all welcome virtual meetings. They have already become essential partners with, and sometimes attractive alternatives to, in‐person events. The science goes on, and everyone wins!

## CONFLICT OF INTEREST

N. D. and D. J. are employees of Keystone Symposia. M. F. and W. M. are members of the board of directors of Keystone Symposia.

## AUTHOR CONTRIBUTIONS

All authors wrote and edited the paper.
